# Changing US Support for Public Health Data Use Through Pandemic and Political Turmoil

**DOI:** 10.1111/1468-0009.12700

**Published:** 2024-05-13

**Authors:** CASON D. SCHMIT, BRIAN N. LARSON, THOMAS TANABE, MAHIN RAMEZANI, QI ZHENG, HYE‐CHUNG KUM

**Affiliations:** ^1^ School of Public Health Texas A&M University; ^2^ School of Law Texas A&M University; ^3^ Transportation Institute Texas A&M University

**Keywords:** privacy preferences, identifiable data, secondary data use

## Abstract

**Policy Points**

- This study examines the impact of several world‐changing events in 2020, such as the pandemic and widespread racism protests, on the US population's comfort with the use of identifiable data for public health. Before the 2020 election, there was no significant difference between Democrats and Republicans. However, African Americans exhibited a decrease in comfort that was different from other subgroups.
- Our findings suggest that the public remained supportive of public health data activities through the pandemic and the turmoil of 2020 election cycle relative to other data use. However, support among African Americans for public health data use experienced a unique decline compared to other demographic groups.

**Context:**

Recent legislative privacy efforts have not included special provisions for public health data use. Although past studies documented support for public health data use, several global events in 2020 have raised awareness and concern about privacy and data use. This study aims to understand whether the events of 2020 affected US privacy preferences on secondary uses of identifiable data, focusing on public health and research uses.

**Methods:**

We deployed two online surveys—in February and November 2020—on data privacy attitudes and preferences using a choice‐based–conjoint analysis. Participants received different data‐use scenario pairs—varied by the type of data, user, and purpose—and selected scenarios based on their comfort. A hierarchical Bayes regression model simulated population preferences.

**Findings:**

There were 1,373 responses. There was no statistically significant difference in the population's data preferences between February and November, each showing the highest comfort with population health and research data activities and the lowest with profit‐driven activities. Most subgroups’ data preferences were comparable with the population's preferences, except African Americans who showed significant decreases in comfort with population health and research.

**Conclusions:**

Despite world‐changing events, including a pandemic, we found bipartisan public support for using identifiable data for public health and research. The decreasing support among African Americans could relate to the increased awareness of systemic racism, its harms, and persistent disparities. The US population's preferences support including legal provisions that permit public health and research data use in US laws, which are currently lacking specific public health use permissions.

Privacy and data protection are urgent issues for federal and state legislatures. In 2022, 29 states introduced a total of 60 comprehensive privacy bills, and five states have enacted similar laws.^1^ In 2022, Congress made substantial progress on a comprehensive federal privacy law, the American Data Privacy and Protection Act.^2^ These legislative efforts are responses to perceived deficiencies in the current legal patchwork of the state and federal privacy and data protection laws.^3^, ^4^, ^5^, ^6^, ^7^ However, these legislative efforts often do not include provisions conducive to existing and future public health data needs, such as public health data‐use exceptions.^6^, ^8^

These omissions are notable given findings of strong and persistent public support for using for public health and research purposes.^5^, ^9^ Wooley and Propst reviewed 88 state and national surveys between 1996 and 2005 and concluded, “the American people want more, not less, research,” and Americans show a “remarkable tolerance for the imperfections” for health‐related research.^10^ Similar support was found by Aitken and colleagues in a 2016 systematic review of public attitudes that uncovers evidence of growing widespread, albeit conditional, support for data sharing and record linkage for research.^11^ Although their evidence demonstrates that data activities that promote social benefits or “the greater good” are generally supported, the literature on public support for data use specifically for public health purposes is less developed prior to the COVID‐19 pandemic.^5^, ^11^ Nonetheless, Schmit and colleagues find evidence for strong public support for using identifiable data for public health applications in a February 2020 nationally representative survey of privacy preferences.^5^ Following that survey, the United States underwent tumultuous events, including the COVID‐19 global pandemic, protests of systemic racism, and increased political polarization.

These unprecedented events amplified awareness of the power, utility, and ubiquity of data analytics in social, governmental, and commercial activities. Big data and technology were used to combat COVID‐19 through disease surveillance and countermeasures, such as contact tracing efforts and symptom‐and‐treatment recognition, with mixed results and notable deficiencies.^12^, ^13^, ^14^, ^15^ Surveillance tactics were also controversially deployed to monitor the social justice protests and unrest that followed the killing of George Floyd.^16^ Throughout 2020, a heavily politicized social climate around such major events increased public awareness and scrutiny of these and other data uses, while contending with unprecedented misinformation.^17^, ^18^ Notably, the politicization and conspiracy theories that proliferated during 2020 likely contributed to alarming increases in threats against public health officials.^19^ These and other notable 2020 events raise legitimate questions about whether our previous findings concerning the public's data‐use preferences for public health remain the same.

The purpose of this paper is twofold. First, we examine whether US residents’ privacy preferences (about which of their identifiable personal data should be used, by whom, and for what purposes) changed during impactful events in 2020. Second, we conduct subgroup analysis to identify potential differences in attitudes toward data use and privacy across several subpopulations, including race, age, gender, income, education, geographic region, and political affiliation. We deployed our initial survey in February 2020, prior to the World Health Organization's declaration that COVID‐19 was a pandemic. We collected data for the second survey in November 2020, after which we assessed changes in population and subpopulation attitudes toward data use and privacy.

A rare opportunity, this paper explores whether the public's data‐use preferences persist when faced by events that might bring them into doubt (e.g., COVID‐19, Black Lives Matter protests). Thus far, the academic literature on the topic has been limited and has not examined the effect that these events have had on the public's preferences for the use of their data.^20^, ^21^, ^22^, ^23^ We hypothesized we would observe changes in overall preferences for using data—specifically changes in preferences relating to public health data uses—and see differences among racial and political‐affiliation subgroups.

## Methods

### Study Design and Recruitment

**Figure 1:**
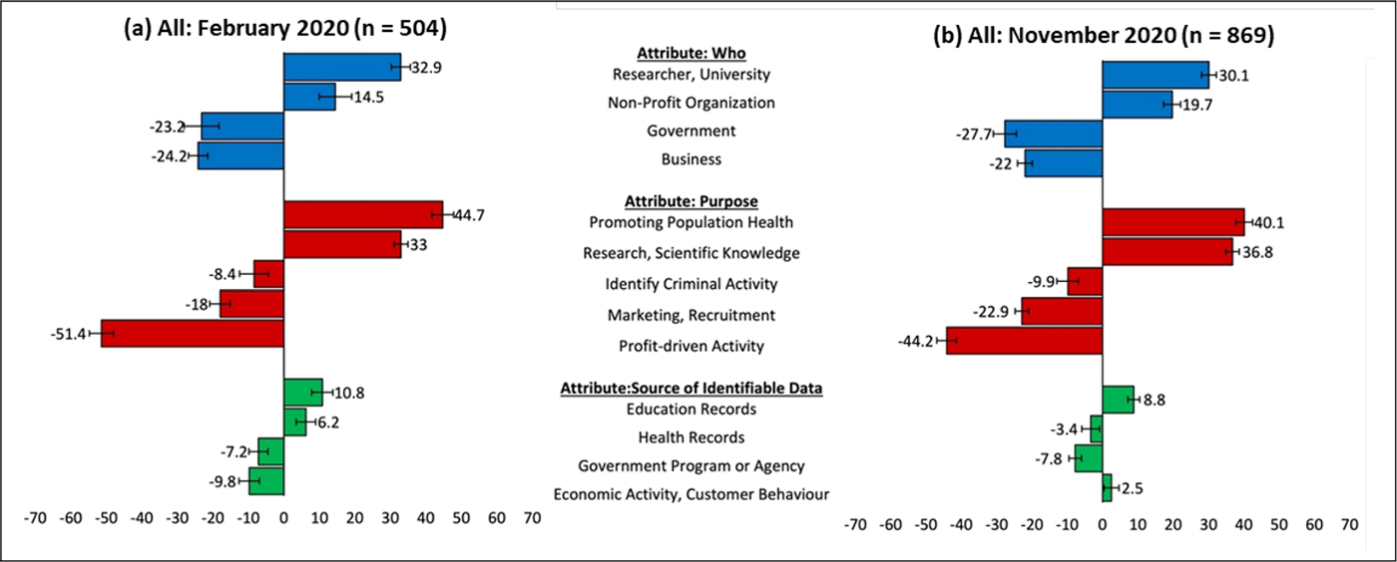
Relative Importance Scores (%) and 84% Confidence Intervals for All Participants’ Preferences for Secondary Uses of Identifiable Data (February and November 2020) [Colour figure can be viewed at wileyonlinelibrary.com]

We used choice‐based–conjoint analysis to measure the preferences of the US public with two online surveys in February 2020 and November 2020, including comfort and preferences for reuse of identifiable data for different purposes without expressed consent. The three attributes for the choice‐based–conjoint questions were based on the following: 1) “who” was using the identifiable data (e.g., university, government, business), 2) the “source” of identifiable data (e.g., health, education, transactions), and 3) the “purpose” of using data (e.g., research, identifying criminal activity, marketing).^5^, ^6^ We selected four to five levels for each attribute to correspond to various legal provisions that permit or restrict data reuse and to identify nuance within categories (e.g., business vs. nonprofit organization), which resulted in 80 (4 × 4 × 5) different scenarios comprised of different attribute levels, listed in Figure 1.^24^ Of those scenarios, we excluded eight as implausible or likely to confuse survey respondents, leaving 72 scenarios.^5^

Because it is not feasible to present all possible combinations of each scenario to participants, we randomly generated subsets of all combinations using a fractional‐factorial design sufficient to obtain robust and meaningful differences in preferences.^5^ The generation resulted in 72 choice sets, with each set consisting of 12 pairs of data‐use scenarios that would allow for simulating participant preferences in the full space of data‐use scenario permutations.^5^ Each participant was randomly assigned to respond to one of the choice sets and selected the data‐use scenario with which they were most comfortable for each of their assigned 12 scenario pairs. Both surveys also measured baseline privacy attitudes using the validated concern for information privacy (CFIP) instrument.^25^ The CFIP has 15 seven‐point Likert scale questions and provides a composite score with four subscales for privacy attitudes. Higher scores indicate higher concern for privacy.^25^

We collected the following demographic information in both administrations: age category (six levels), gender (three levels), race (five levels), income (five levels), educational attainment (five levels), US region (four levels), health insurance coverage (six levels), existence of any chronic condition, use of a health care provider in last year, and use of an emergency room in last year.

The significant events of 2020 compelled us to redeploy the survey with some enhancements. Specifically, we were interested to see whether our prior findings (i.e., strong public support for the use of identifiable data to promote population health and science or research and strong concerns regarding government and businesses’ use of data for profit‐driven and marketing purposes) might have changed because of the COVID‐19 pandemic or any of the other significant 2020 events.^5^ The November survey included additional questions to provide additional context. For instance, only the November survey contained questions on political affiliation and attitudes. Additionally, in the November survey, we added several open‐ended questions that allowed participants to express what was most important to their decisions concerning with what they were or were not comfortable and whether they found any data‐sharing scenarios to be unacceptable.

We used Dynata, a recognized leader in market research that specializes in nationally representative sampling to recruit potential participants.^26^ Dynata independently recruits participants via email from its panel to meet desired recruitment targets. Individuals within Dynata's proprietary panel of potential respondents are screened to ensure accurate demographic information. We limited our recruitment to adult (≥18 years) US residents, and we sought to balance the sample on six targets using population characteristics from the Census Bureau (gender, race/ethnicity, age, education, household income, and region) where possible. We limited our sample to those participants who responded before the declaration of the winner of the 2020 presidential election to ensure that our sample reflected the climax of social politicization.

### Statistical Analysis

Several statistical models have been used to analyze discrete choice experiments.^27^, ^28^ We chose a hierarchical Bayes multinomial logit model to infer utility and other key parameter values. Specifically, we relied on the R package ChoiceModelR that used an efficient Monte Carlo Markov chain^29^ simulation algorithm to generate posterior draws for model parameters. We generated 10,000 posterior draws for each parameter and constructed 84% confidence intervals for comparisons of utilities between the two independent surveys. Prior work shows that nonoverlapping 84% confidence intervals for two samples approximates statistical significance at the *p* = 0.05 level.^28^, ^30^, ^31^ Consequently, we considered two 84% confidence intervals that do not overlap statistically different.^28^, ^30^, ^31^

This approach measures both the relative importance of each attribute—who was using the identifiable data versus the source of the data versus the purpose of the use—and the relative acceptability of each level in each attribute (e.g., university or researcher vs. nonprofit vs. business vs. government). Thus, if the size of the relative importance score values (positive or negative) for “purpose” are greater, the size of the relative importance score values for “who” are using and/or the “source” will be smaller. Within the attributes, the values for all levels add to zero so that when a level has a large positive value there must be corresponding negative value distributed among other levels. Greater differences between positive and negative relative importance scores indicate more divergent opinions on attribute levels. Importantly, some caution is necessary in interpreting the results because it is difficult to determine whether an observed change in a relative importance score is independently meaningful or if it is an epiphenomenon related to an observed change in another attribute or level.

## Results

For the February and November surveys, 687 and 1,533 participants responded to the recruitment, of which 665 and 1,396 agreed to participate, respectively. In total, 1,373 individuals completed the survey: 504 in February 2020 and 869 in November 2020. Overall, our census sampling targets for age, gender, race, income, education, and census region were generally met for both surveys (Table 1). Health insurance coverage was not a target we aimed for, but the sample paralleled similar rates published by the US Census Bureau. Over one‐third of respondents reported having at least one chronic health condition (February = 35.9%, November = 34.3%), almost one‐half of respondents used a health provider within the past year (February = 48.6%, November = 48.8%), and roughly one‐fifth of respondents visited an emergency department in the last year (February = 20%, November = 14.7%).

**Table 1:** Demographics of Respondents for the Surveys Deployed in February 2020 and November 2020

Participant Characteristics	February (*n* = 504)	November (n = 869)	Sample Target (%)
Age categories (y), *n* (%)			
18‐24	41 (8.1)	80 (9.21)	13.1
25‐34	75 (14.9)	162 (18.64)	17.5
35‐44	100 (19.8)	167 (19.22)	17.5
45‐54	101 (20.0)	168 (19.33)	19.2
55‐64	68 (13.5)	134 (15.42)	15.6
≥65	89 (17.7)	153 (17.61)	17.2
Missing data	30 (5.95)	5 (0.58)	—
Gender, *n* (%)			
Male	224 (44.4)	376 (43.27)	48.5
Female	278 (55.2)	487 (56.04)	50.5
Other/prefer not to answer	2 (0.4)	6 (0.69)	—
Race categories, *n* (%)			
White	315 (62.5)	635 (73.07)	63.7
African American	77 (15.3)	118 (13.58)	12.2
Hispanic	51 (10.1)	49 (5.64)	16.4
Asian American	46 (9.1)	49 (5.64)	4.7
Other	15 (3.0)	18 (2.07)	3.0
Income categories ($), *n* (%)			
≥$20,000	103 (20.4)	148 (17.03)	19.9
20,000‐49,999	149 (29.6)	254 (29.23)	30.6
50,000‐99,999	137 (27.2)	285 (32.80)	29.1
100,000‐149,999	67 (13.30)	116 (13.35)	12.0
≤150,000	48 (9.5)	66 (7.59)	8.3
Educational level, *n* (%)			
High school or less	172 (34.1)	292 (33.60)	32.0
Some college completed	99 (19.6)	160 (18.41)	19.0
College degree	191 (37.9)	304 (34.98)	31.0
Master degree	37 (7.3)	85 (9.78)	—
PhD/doctoral	5 (1)	28 (3.22)	—
Region, *n* (%)			
Midwest	95 (18.8)	171 (19.68)	22.0
Northeast	126 (25)	186 (21.40)	18.2
South	174 (34.5)	346 (39.82)	36.2
West	109 (21.6)	166 (19.10)	23.6
Health insurance coverage, *n* (%)			
Private	169 (33.5)	309 (35.56)	64.7
Medicare	112 (22.2)	188 (21.63)	17.7
Medicaid	83 (16.5)	144 (16.57)	17.9
Uninsured	52 (10.3)	88 (10.13)	8.5
Veterans Affairs/TRICARE	10 (2)	21 (2.42)	3.6
Multiple	78 (15.5)	119 (13.69)	14.5
Any chronic condition, *n* (%)			
Yes	181 (35.9)	298 (34.29)	—
Use of health care provider in the past year, *n* (%)			
Yes	245 (48.6)	424 (48.8)	—
≥1 emergency department visit in the last year, *n* (%)			
Yes	101 (20)	128 (14.7)	—

Generally, CFIP scores showed no marked changes between February and November 2020 (Table A1). This section presents our principal aggregate findings and then considers two subgroups: African Americans and Democrats versus Republicans.

### Principal Findings

Figure 1 presents the relative importance for the different attribute levels across the two surveys for all participants. Values are relative within the attribute, and positive and higher values indicate stronger preference for potential data reuse.

Generally, the relative importance scores between the February and November samples maintained stable patterns with only a few statistically significant differences (Figure 1). Relative importance scores for the “who” attribute remained relatively stable in 2020 with no statistically significant differences for any attribute level between February and November. Universities/researchers and nonprofits had the highest relative acceptability, and governments and businesses had the lowest. Similarly, there was stability in the order of relative preferences for the different options for data‐use “purpose.” Public health and research remained the most supported data uses, followed by identifying criminal activity, marketing/recruitment, and profit‐driven activities. Despite the stability in preference order, there were statistically significant differences in relative importance values for data‐use purposes (i.e., their 84% confidence intervals were not overlapping) for research, marketing, and profit‐driven activities. The relative importance of data type was consistently lower than the other attributes in February and November. Education data remained the most supported data types, and government program data maintained low relative importance scores. However, we observed a statistically significant decrease in relative acceptability for health data and a statistically significant increase in relative acceptability for economic activity data.

**Figure A1:**
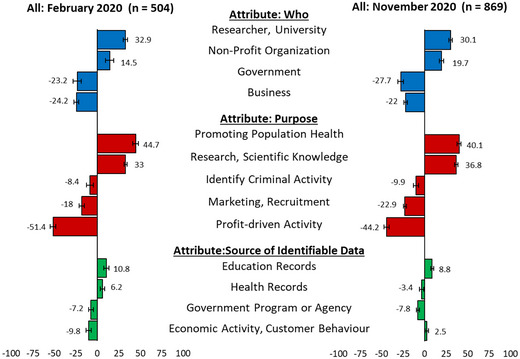
Relative importance scores (%) and 84% confidence intervals for all participants’ preferences for secondary uses of identifiable data (February and November 2020) [Colour figure can be viewed at wileyonlinelibrary.com]

**Figure A2:**
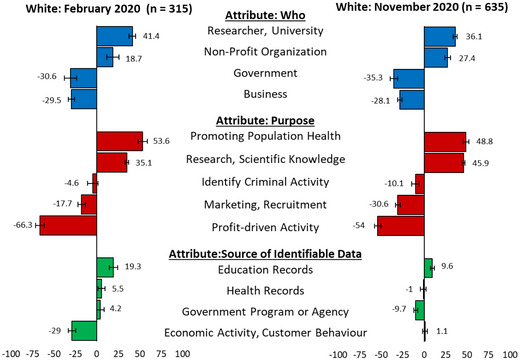
Relative importance scores (%) and 84% confidence intervals for White participants’ preferences for secondary uses of identifiable data (February and November 2020) [Colour figure can be viewed at wileyonlinelibrary.com]

**Figure A3:**
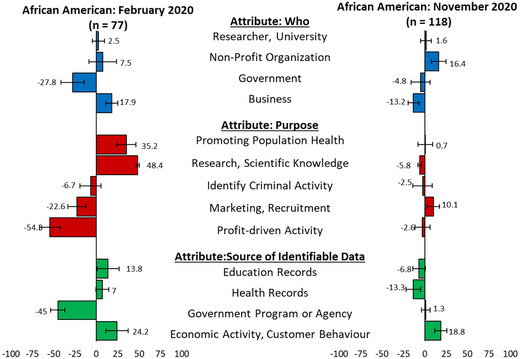
Relative importance scores (%) and 84% confidence intervals for African American participants’ preferences for secondary uses of identifiable data (February and November 2020) [Colour figure can be viewed at wileyonlinelibrary.com]

**Figure A4:**
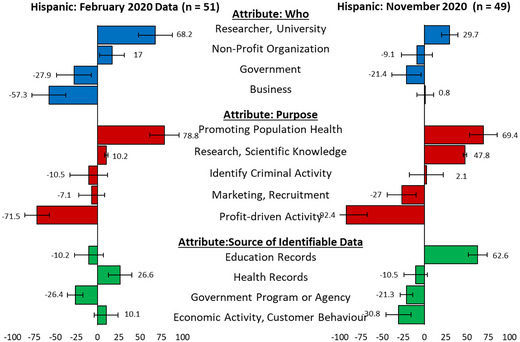
Relative importance scores (%) and 84% confidence intervals for Hispanic participants’ preferences for secondary uses of identifiable data (February and November 2020) [Colour figure can be viewed at wileyonlinelibrary.com]

**Figure A5:**
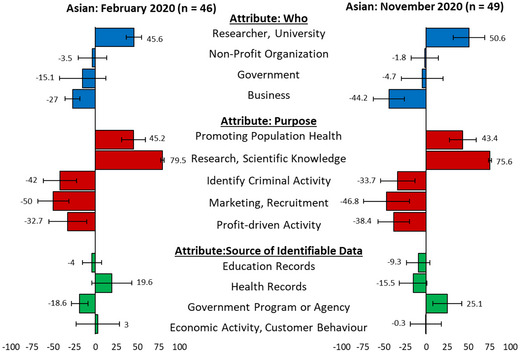
Relative importance scores (%) and 84% confidence intervals for Asian participants’ preferences for secondary uses of identifiable data (February and November 2020) [Colour figure can be viewed at wileyonlinelibrary.com]

**Figure A6:**
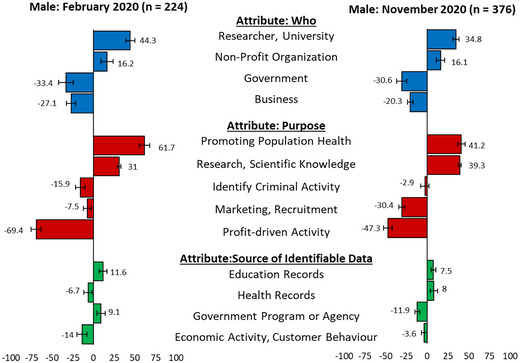
Relative importance scores (%) and 84% confidence intervals for Male participants’ preferences for secondary uses of identifiable data (February and November 2020) [Colour figure can be viewed at wileyonlinelibrary.com]

**Figure A7:**
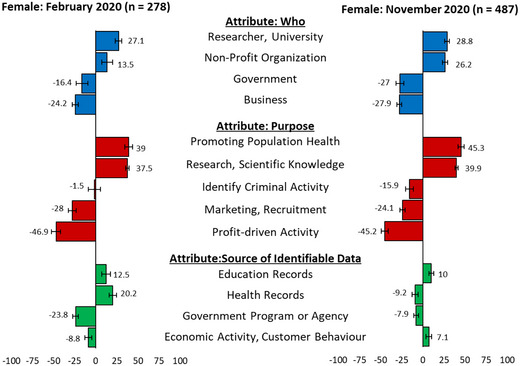
Relative importance scores (%) and 84% confidence intervals for Female participants’ preferences for secondary uses of identifiable data (February and November 2020) [Colour figure can be viewed at wileyonlinelibrary.com]

**Figure A8:**
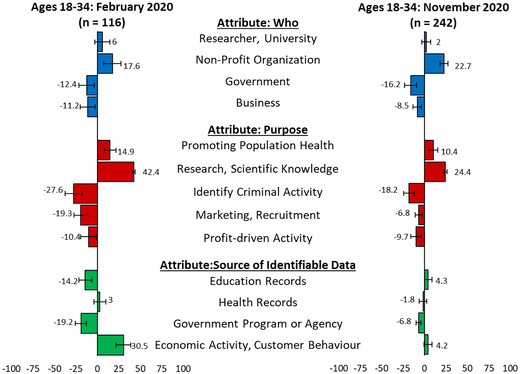
Relative importance scores (%) and 84% confidence intervals for 18–34 years old participants’ preferences for secondary uses of identifiable data (February and November 2020) [Colour figure can be viewed at wileyonlinelibrary.com]

**Figure A9:**
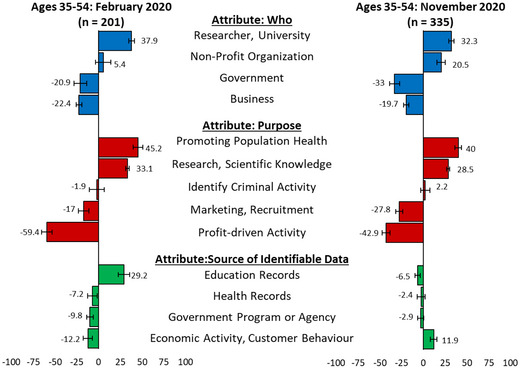
Relative importance scores (%) and 84% confidence intervals for 35–54 years old participants’ preferences for secondary uses of identifiable data (February and November 2020) [Colour figure can be viewed at wileyonlinelibrary.com]

**Figure A10:**
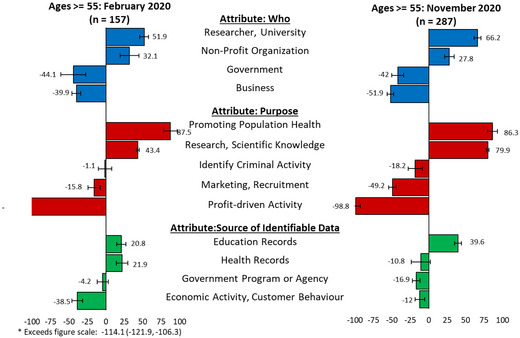
Relative importance scores (%) and 84% confidence intervals for 55 years old and over participants’ preferences for secondary uses of identifiable data (February and November 2020) [Colour figure can be viewed at wileyonlinelibrary.com]

**Figure A11:**
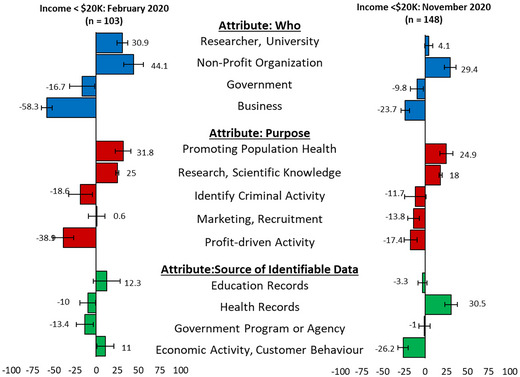
Relative importance scores (%) and 84% confidence intervals for participants with income less than $20,000 preferences for secondary uses of identifiable data (February and November 2020) [Colour figure can be viewed at wileyonlinelibrary.com]

**Figure A12:**
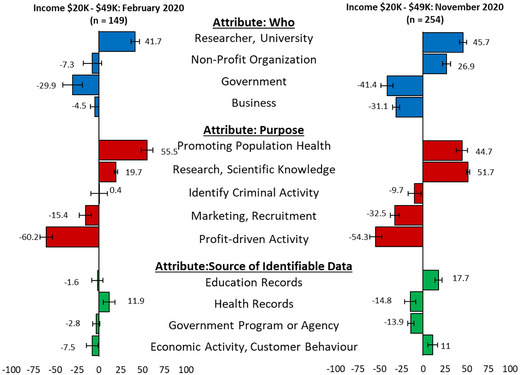
Relative importance scores (%) and 84% confidence intervals for participants with income between $20,000 ‐$49,000 preferences for secondary uses of identifiable data (February and November 2020) [Colour figure can be viewed at wileyonlinelibrary.com]

**Figure A13:**
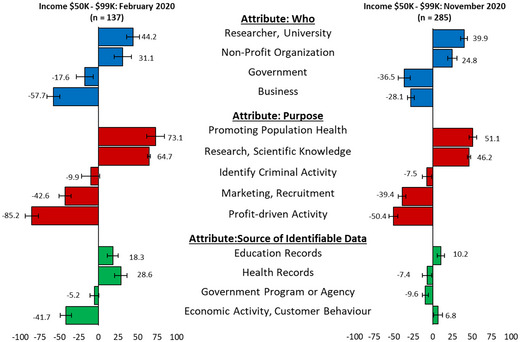
Relative importance scores (%) and 84% confidence intervals for participants with income between $50,000 ‐$99,000 preferences for secondary uses of identifiable data (February and November 2020) [Colour figure can be viewed at wileyonlinelibrary.com]

**Figure A14:**
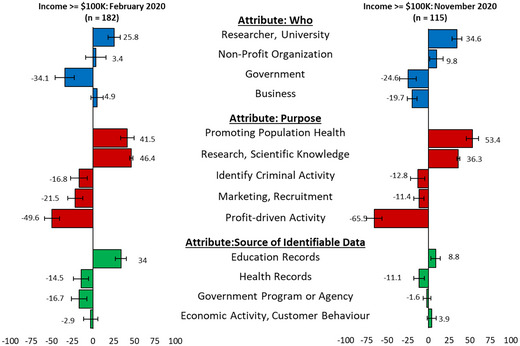
Relative importance scores (%) and 84% confidence intervals for participants with income $100,000 or more preferences for secondary uses of identifiable data (February and November 2020) [Colour figure can be viewed at wileyonlinelibrary.com]

**Figure A15:**
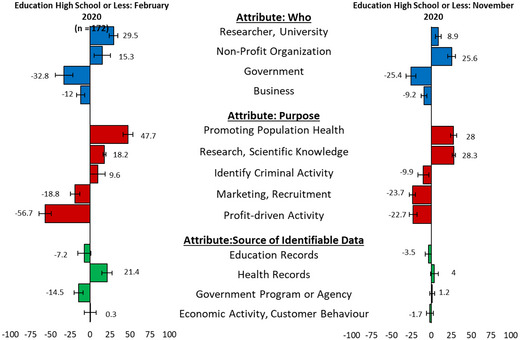
Relative importance scores (%) and 84% confidence intervals for participants with high school or less education for secondary uses of identifiable data (February and November 2020) [Colour figure can be viewed at wileyonlinelibrary.com]

**Figure A16:**
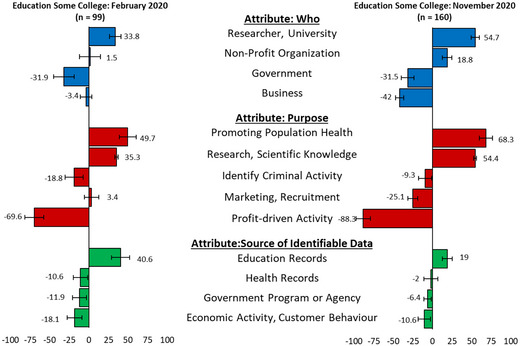
Relative importance scores (%) and 84% confidence intervals for participants with some college education for secondary uses of identifiable data (February and November 2020) [Colour figure can be viewed at wileyonlinelibrary.com]

**Figure A17:**
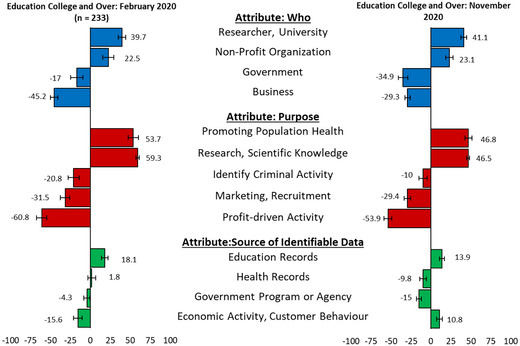
Relative importance scores (%) and 84% confidence intervals for participants with college and over education for secondary uses of identifiable data (February and November 2020) [Colour figure can be viewed at wileyonlinelibrary.com]

**Figure A18:**
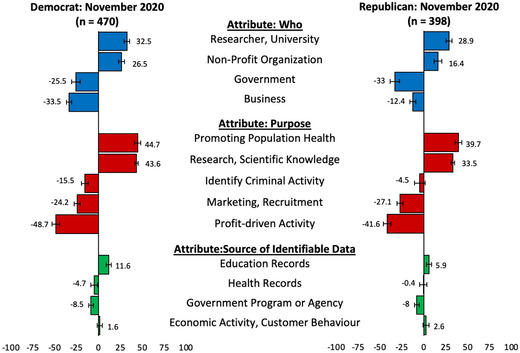
Relative importance scores (%) and 84% confidence intervals for Democrat and Republican participants’ preferences for secondary uses of identifiable data (November 2020) [Colour figure can be viewed at wileyonlinelibrary.com]

The relative importance score distributions for nearly all subgroups were similar to the general population's distribution in the February sample, which indicates a general preference consensus across the subgroups. However, our results show less consensus across the subgroups in the November sample. Some subgroups experienced more significant perception shifts than others. Appendix 2 (Table A2) contains the overall and all subgroup analyses with 84% confidence intervals. Appendix 3 (Figures A1, A2, A3, A4, A5, A6, A7, A8, A9, A10, A11, A12, A13, A14, A15, A16, A17, A18)provides figures comparing subgroups across the February and November samples. A notable change is visible among African Americans, and we noted in the November administration that those identifying as Democrats and Republicans showed some significant differences from each other as well.

#### African Americans

**Figure 2:**
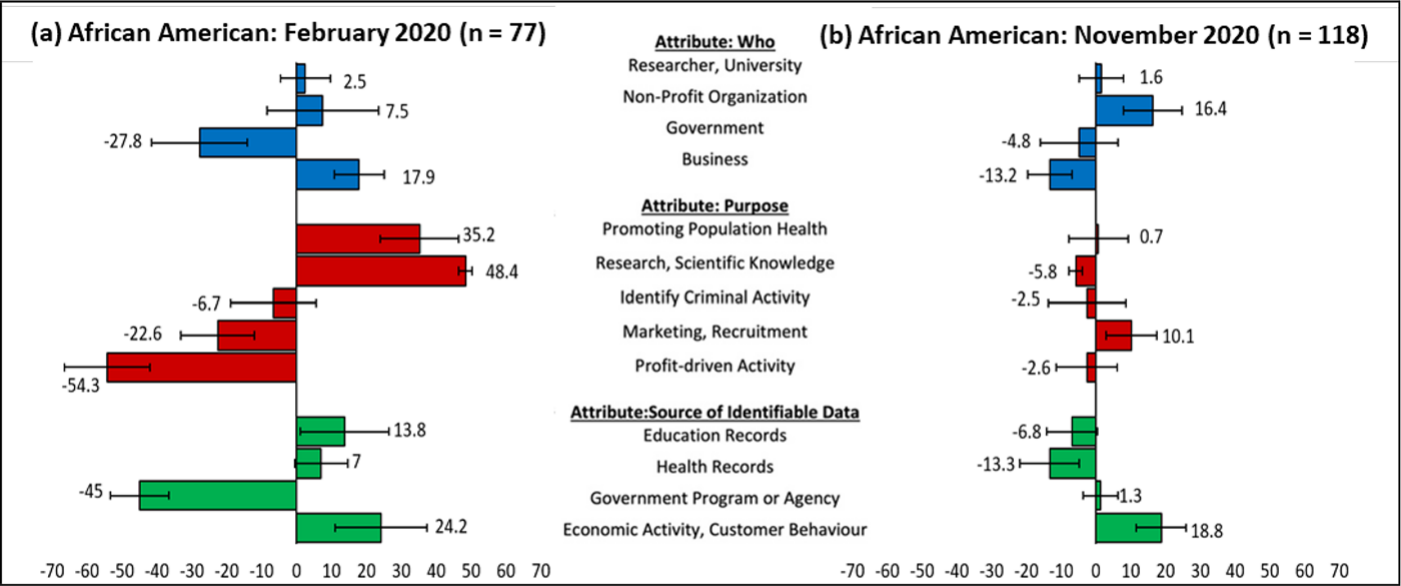
Relative Importance Scores (%) and 84% Confidence Intervals for African American Participants’ Preferences for Secondary Uses of Identifiable Data (February and November 2020) [Colour figure can be viewed at wileyonlinelibrary.com]

As Figure 2 shows, African American participants exhibited notable differences between February and November 2020. The African Americans participants showed the strongest negative shift in relative preference for using data for research (48.4% to −5.8%) and promoting public health purposes (35.2%‐0.7%). Conversely, the Africans American participants’ relative preference of using data for profit‐driven (−54.3% to −2.6%) and marketing activities (22.6%‐10.1%) improved to counterbalance. The African Americans participants’ relative preference for businesses using data significantly declined (17.9% to −13.2%), whereas the relative preference for the government using data increased significantly (−27.8% to −4.8%). The African Americans participants’ relative preferences for using government sources of data had a statistically significant increase (−45% to 1.3%), whereas using health and education data sources both significantly decreased (health = 7% to −13.3%, education = 13.8% to −6.8%).

A thematic analysis of the open‐ended questions in the November survey revealed trust and the perceived value or social benefit of the data use as reoccurring themes. Related to the perceived value or social benefit of the data use, many open‐ended responses reflected skepticism for profit‐driven activities or activities intended to detect criminal behavior. Related to trust, many African American respondents’ open‐ended responses reflected distrust of government and businesses. One African American respondent stated:

Historically information gathered by the government works against those who are not wealthy. For example the Tuskegee Experiment, where African American men were used as test subjects for syphilis research were all very poor. Examples like this in history always uses the disenfranchised and never the wealthy. Some of the wealthy feel that this experiment was moral. I would like to see if they feel the same way if the experiment was done on them or their loved ones. Finally the wealthy reap the benefits with no risk or worry. I will trust the government having my info when rich and poor are experimented on, until then I will remain skeptical.

#### Political Affiliation

**Figure 3:**
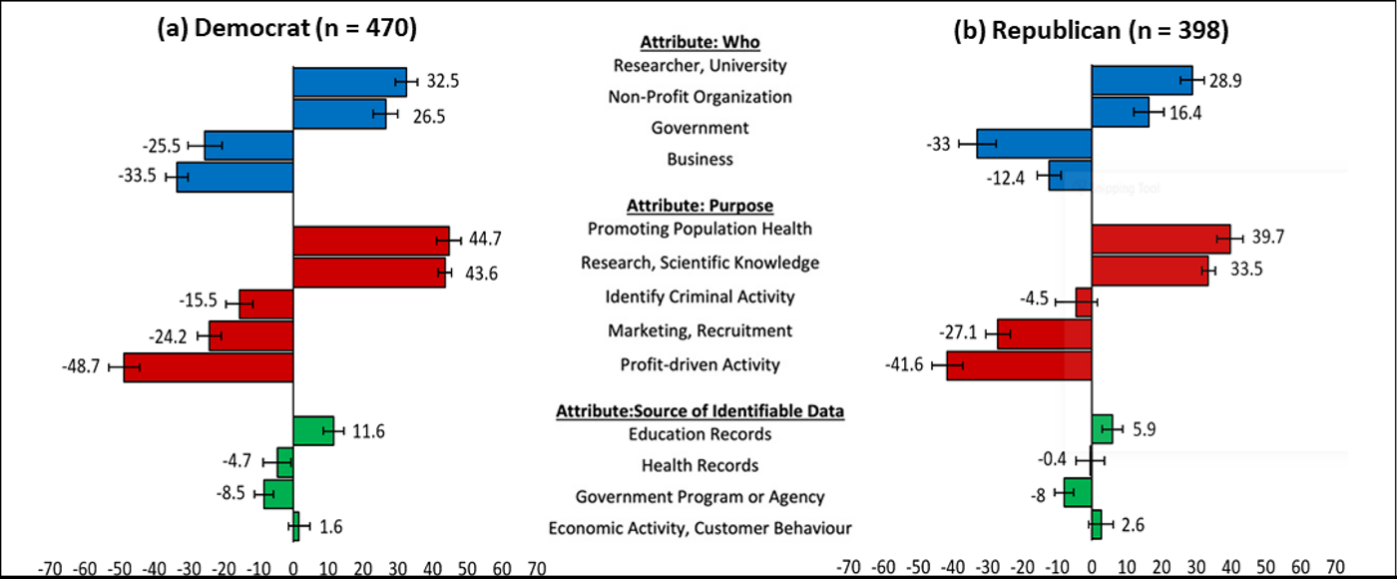
Political Party Subgroups’ Relative Importance Scores and 84% Confidence Intervals for Secondary Use of Data, November 2020 [Colour figure can be viewed at wileyonlinelibrary.com]

In the November survey, there was a general consensus in preferences across respondents, subgrouped by political affiliation. Democrat and Republican respondents’ most split preferences were concerning the “purpose” of data‐use attribute in comparison to “who” was using the data or the “source” of the identifiable data. Within the proposed data‐use attribute, there was a statistically significant difference between Democrats and Republicans in their relative importance preference for using data for research (Democrats = 43.6%, Republicans = 33.5%) and to identify criminal activity (Democrats = −15.5%, Republicans = −4.5%). Within the who is using the data attribute, there was a statistically significant difference between Democrats and Republicans in their relative importance preference for businesses (Democrats = −33.5%, Republicans = −12.4%) and nonprofit organizations (Democrats = 26.5%, Republicans = 16.4%) to use identifiable data (Figure 3).

## Discussion

### Principal Finding: Public Support for Public Health Use of Data Remained Strong

Here, we surveyed US respondents’ data‐use preferences to examine to what extent the impactful events of 2020 changed privacy preferences. Our findings, overall, show that the events of 2020 did not dent strong public support for public health use of data. Notably, the results include very strong support for public health and research data use—relative to other use scenarios—across the political spectrum for both self‐identified Democrats and Republicans. These findings provide a contrast to the high‐profile threats to public health officials that occurred during the height of the pandemic.^19^ Participants in the November sample were still the most comfortable with university‐affiliated researchers or nonprofit organizations using data to promote population health and science or research. In contrast, the participants were the least comfortable with the government and businesses using data for profit‐driven and marketing purposes.

These results were generally consistent across all subgroups among survey administrations, indicating general agreement and uniformity among respondents across subgroup stratification and across time. The consistency suggests that the world‐changing events of 2020 did not dramatically shift attitudes on the reuse of identifiable data without consent. Nevertheless, we found a few noteworthy changes in preferences. In the following sections, we provide potential explanations for and discuss the future implications of our findings.

### African American Participants Demonstrate the Most Dramatic Changes by Demographic Group

African American respondents, in comparison to any other demographic subgroups, demonstrated the most substantial changes in privacy preferences across the three attributes. Although their February scores closely resembled the national sample—with similar strong support for public health and research and low support for profit‐driven activities—the November results show a significant shift toward agnosticism on most measures. The design limits causal inferences from our results. For instance, confounding variables are abundant given the confluence of traumatic, galvanizing, and noteworthy events. As a result, changes in relative importance scores could have alternative explanations (see Figure 1).

Nonetheless, the zero‐net change in baseline privacy concerns (CFIP scores) suggests that the changes in the relative importance scores for African American participants could reflect a change in concerns about the specific activities in our data‐use scenarios (e.g., racial exploitation in research, racial public health disparities) rather than shifts in overall privacy concerns.

There are numerous potential factors and events between February and November 2020 that are unique to the experiences of Black people and may have contributed to increased mistrust of certain activities in African American perceptions. We propose three possible nonexclusive, and potentially synergistic, explanations.

First, we note that awareness of systemic racism, particularly against African American individuals, crescendoed in 2020. Multiple high‐profile killings of unarmed African American people reignited intense protests and social justice awareness. The literature has shown increased mistrust in institutions following these protests.^32^, ^33^, ^34^ In fact, privacy was a large concern of the Black Lives Matter protesters. Many protest organizers distributed privacy guides that contained recommendations, such as disabling phone biometrics and using encrypted messaging applications.^16^

Second, historical and present abuses and disparities have led to lingering mistrust in research and medical activities within African American communities. This mistrust in medical and scientific institutions is well documented.^35^, ^36^ Notably, this effect was not apparent in the February survey. However, it is possible that the increased awareness of historical and current systemic racism—as previously discussed—and the disparities and inequities of the COVID‐19 pandemic—discussed next—amplified mistrust of researchers and health officials in the November sample.^37^

Finally, African Americans had higher social, economic, and health disparities related to the COVID‐19 pandemic, including higher infections and mortality.^38^ Moreover, disparities led to intense public calls for increased involvement of African American individuals in the testing and administration of new vaccines. This need could have raised legitimate questions and concerns among African American communities regarding exploitation, including how data were collected, analyzed, reported, and ultimately used without any significant addressment as a health disparity during the pandemic. The open‐ended responses from African American respondents provide some support for these possible explanations. Some responses reflected themes of trust and, specifically, mistrust in government. One African American participant's explanation of their responses (previously quoted) reflected elements of each of these three possible explanations for the data‐use preferences of African American respondents in the November 2020 survey. However, caution is warranted in reading too much into the open‐ended responses because not all respondents provided detailed explanations of their answers. Furthermore, the November 2020 survey did not include open‐ended questions, so differences among the samples can not be assessed.

These possible explanations offer starting points for continued discussion and further inquiry into why African American people's data preferences changed over the course of several months in 2020. Additional research should focus on other factors or relevant events that could explain the shift in perceptions or propose a more comprehensive model that does so. However, we observe that trust in public health and research enterprises is fragile. Ongoing injustice might reopen old scars, leading to renewed mistrust. Public health data stewards and researchers should be keenly aware that their social license to use data for public health activities is precarious and vulnerable to injustices baked into structural institutions and persist in social environments.

### Incongruence of National Privacy Legislation and Public Preferences

At the population level, across political parties and within nearly all subgroups, there is strong support for using identifiable data for public health and research purposes without an individual's consent. Our results advance the notion that the public's data‐use preferences are in favor of legal provisions within data protection and privacy laws that permit research and public health data use. However, these provisions—particularly for public health data use—are typically rare in US laws, with some notable exceptions (e.g., the Health Insurance Portability and Accountability Act).^3^, ^4^ Consequently, our findings present a striking incongruence between public preferences for research and data use and US laws.

For example, the Family Educational Rights and Privacy Act of 1974 (FERPA) protects education data. Education is one of the most potent social determinants of health, so these data have tremendous public health value. Nonetheless, FERPA's data‐use provisions do not include exceptions for public health or health‐related research. Notably, these were the two data uses that the US public were most comfortable with across both samples, showing how current laws often impede socially acceptable and beneficial data use. This misalignment persists in contemporary legislative efforts to address perceived US privacy law deficiencies, which sometimes erect barriers to modernizing public health informatics by restricting valuable social determinants data from being used for public health purposes.^2^, ^8^

These findings are well timed within the current US public health data modernization initiative. As part of this initiative, federal and state policies considering the collection and use of data to promote population health are being reevaluated. Moreover, there are considerable investments in public health information technology infrastructure. Laws that restrict the use of data that pertains to social determinants of health place an artificial ceiling on what public health informatics can learn about the causes of poor health and social well‐being.^8^ Our findings could inform a recalibration of existing policies to support more public health and research use of available data, uses that the US public are far more comfortable with than existing private‐sector data practices.

### Limitations

The survey is not exhaustive of all data‐use scenarios; therefore, the measured participants’ preferences are relative to the 72 provided scenarios.^5^ Moreover, this design measured participants’ preferences rather than acceptability, which means that a participant's least preferred scenario could still be acceptable or the most preferred scenario might still be unacceptable. Additionally, although our sample size permits national and census‐region comparisons, it is not sufficiently large for smaller geographic comparisons (e.g., among states). Moreover, although the two samples are relatively similar, there were some differences in the composition of race and education level.

## Conclusions

It is vital for elected officials to understand the policy preferences of their constituents, so they protect individual privacy and promote public benefit. Despite a momentously eventful year such as 2020, the US public demonstrates continued support for data use to promote population health and research. Notably, we found this was bipartisan support despite a remarkably politically polarizing year in 2020.

Common data‐use exceptions for public health and research across federal and state data protection laws are needed. Inconsistent treatment of public health and research in laws creates challenges for public health professionals and researchers and impedes scientific discovery and benefits for the public good. Our findings provide new support for allowances for public health and research data use in new privacy legislation.

The data from these surveys are not publicly available but can be made available from the corresponding author upon reasonable request.

## Funding/Support

This study received internal funding from Texas A&M University and no external funding.

## Conflict of Interest Disclosures

The authors have no conflict of interest to declare.

## Appendix

**Table A.1:** Respondents’ Concerns for Information Privacy Scores by subgroups for the surveys deployed in February and November 2020

Month	Participant characteristics	Subscale 1	Subscale 2	Subscale 3	Subscale 4	Overall CFIP
**Feb**	All (N = 504)	5.49	5.67	6.03	6.06	5.8
**Nov**	All (N = 869)	5.54	5.67	5.99	6.02	5.79
	**Race categories, N**					
**Feb**	White (N = 315)	5.55	5.83	6.24	*6.28	5.95
**Nov**	White (N = 635)	5.59	5.76	6.14	*6.17	5.9
**Feb**	African American (N = 77)	5.27	5.39	5.6	5.61	5.46
**Nov**	African American (N = 118)	5.36	5.42	5.53	5.54	5.46
**Feb**	Hispanic (N = 51)	5.38	5.38	5.74	5.78	5.56
**Nov**	Hispanic (N = 49)	5.53	5.44	5.53	5.65	5.53
**Feb**	Asian (N = 46)	5.5	5.4	5.6	5.59	5.52
**Nov**	Asian (N = 49)	5.44	5.6	5.63	5.78	5.6
	**Gender categories, N**					
**Feb**	Male (N = 224)	5.52	5.66	6.03	**6.12	5.81
**Nov**	Male (N = 376)	5.5	5.58	5.87	**5.95	5.71
**Feb**	Female (N = 278)	5.47	5.69	6.06	6.03	5.8
**Nov**	Female (N = 487)	5.59	5.75	6.1	6.09	5.87
	**Age categories (years), N**					
**Feb**	Age 18 ‐ 34 (N = 116)	5.34	5.29	5.69	5.64	5.48
**Nov**	Age 18 ‐ 34 (N = 242)	5.34	5.38	5.54	5.57	5.45
**Feb**	Age 35 ‐ 54 (N = 201)	5.48	5.71	6.02	6.06	5.8
**Nov**	Age 35 ‐ 54 (N = 335)	5.53	5.66	5.94	5.96	5.76
**Feb**	Age > = 55 (N = 157)	5.65	6.01	6.42	6.47	6.12
**Nov**	Age > = 55 (N = 287)	5.75	5.97	6.47	6.5	6.15
	**Income categories, N**					
**Feb**	Income 20K or less (N = 103)	5.25	5.47	6	5.85	5.63
**Nov**	Income 20K or less (N = 148)	5.39	5.52	5.79	5.77	5.61
**Feb**	Income 20K ‐ 49K (N = 149)	5.44	5.73	5.93	6.07	5.77
**Nov**	Income 20K ‐ 49K (N = 254)	5.5	5.72	6.06	6.06	5.82
**Feb**	Income 50K ‐ 99K (N = 137)	5.47	5.67	6.09	6.06	5.81
**Nov**	Income 50K ‐ 99K (N = 285)	5.6	5.68	6.01	6.06	5.82
**Feb**	Income 100K or more (N = 115)	5.79	5.77	6.13	6.22	5.96
**Nov**	Income 100K or more (N = 182)	5.62	5.72	6.01	6.1	5.85
	**Educational level, N**					
**Feb**	High school or less (N = 172)	5.38	**5.79	**6.06	**6.06	5.81
**Nov**	High school or less (N = 292)	5.46	**5.53	**5.85	**5.85	5.66
**Feb**	Some college (N = 99)	5.46	**5.6	*6.02	**6	*5.76
**Nov**	Some college (N = 160)	5.63	**5.99	*6.26	**6.32	*6.03
**Feb**	College and over (N = 233)	5.48	5.6	5.96	5.95	5.8
**Nov**	College and over (N = 417)	5.57	5.65	5.98	6.02	5.79
	**Political Affiliation, N**					
**Nov**	Democrat (N = 470)	5.53	5.71	6.0	6.01	5.81
**Nov**	Republican (N = 398)	5.56	5.63	5.98	6.03	5.8

** P value < 0.05

* P value < 0.1

## Appendix

**Table A.2:** Relative Importance (84% Confidence Interval) Comparison of Data Use Scenario Attributes (who is using the data; purpose of data use; data source) by Race and Political Affiliation for the surveys deployed in February 2020 and November 2020

WHO					
Survey	Group	Researcher, University	Government	Business	NonProfit Organization
Nov	All	30.1 % (27.9 to 32.2)	−27.7 % (−31.1 to −24.4)	−22 % (−24 to −20)	19.7 % (17.3 to 22.1)
Feb	All	32.9 % (30.2 to 35.6)	−23.2 % (−28.1 to −18.3)	−24.2 % (−28 to −20.5)	14.5 % (10 to 19.1)
Nov	White	36.1 % (33.5 to 38.6)	−35.3 % (−39.6 to −31)	−28.1 % (−30.8 to −25.4)	27.4 % (24.1 to 30.7)
Feb	White	41.4 % (37.7 to 45.1)	−30.6 % (−37.6 to −23.6)	−29.5 % (−35.2 to −23.9)	18.7 % (11.7 to 25.8)
Nov	African American	1.6 % (−4.8 to 8)	−4.8 % (−16 to 6.3)	−13.2 % (−21.1 to −5.3)	16.4 % (8 to 24.8)
Feb	African American	2.5 % (−4.7 to 9.6)	−27.8 % (−41.5 to −14.1)	17.9 % (5.6 to 30.1)	7.5 % (−8.5 to 23.4)
Nov	Hispanic	29.7 % (19.8 to 39.5)	−21.4 % (−38.8 to −4)	0.8 % (−16.2 to 17.8)	−9.1 % (−27.3 to 9.1)
Feb	Hispanic	68.2 % (48.1 to 88.2)	−27.9 % (−48.2 to −7.6)	−57.3 % (−71 to −43.6)	17 % (2.3 to 31.7)
Nov	Asian	50.6 % (32 to 69.2)	−4.7 % (−29.6 to 20.2)	−44.2 % (−61.9 to −26.5)	−1.8 % (−17.9 to 14.4)
Feb	Asian	45.6 % (36.7 to 54.5)	−15.1 % (−42.5 to 12.3)	−27 % (−53.4 to −0.6)	−3.5 % (−20.3 to 13.3)
Nov	Democrat	32.5 % (29.3 to 35.6)	−25.5 % (−30.4 to −20.6)	−33.5 % (−36.6 to −30.3)	26.5 % (22.9 to 30)
Nov	Republican	28.9 % (25.6 to 32.3)	−33 % (−38.3 to −27.6)	−12.4 % (−15.8 to −9)	16.4 % (12.1 to 20.7)

Purpose						
Survey	Group	Research, Scientific Knowledge	Identify Criminal Activity	Promoting Population Health	Marketing, Recruitment	Profit‐driven Activity
Nov	All	36.8 % (35 to 38.7)	−9.9 % (−13 to −6.8)	40.1 % (37.8 to 42.5)	−22.9 % (−24.8 to −21)	−44.2 % (−47 to −41.4)
Feb	All	33 % (31.1 to 34.9)	−8.4 % (−12.4 to −4.3)	44.7 % (41.6 to 47.8)	−18 % (−20.8 to −15.1)	−51.4 % (−54.7 to −48)
Nov	White	45.9 % (43.2 to 48.5)	−10.1 % (−14.3 to −5.8)	48.8 % (45.7 to 51.9)	−30.6 % (−33.2 to −28)	−54 % (−57.7 to −50.3)
Feb	White	35.1 % (31.7 to 38.5)	−4.6 % (−10.7 to 1.5)	53.6 % (48.4 to 58.8)	−17.7 % (−22 to −13.4)	−66.3 % (−71.5 to −61.1)
Nov	African American	−5.8 % (−9.7 to −1.9)	−2.5 % (−13.7 to 8.7)	0.7 % (−7.8 to 9.2)	10.1 % (2.9 to 17.4)	−2.6 % (−11.3 to 6.2)
Feb	African American	48.4 % (43.6 to 53.2)	−6.7 % (−19 to 5.6)	35.2 % (24 to 46.5)	−22.6 % (−33.2 to −12)	−54.3 % (−66.5 to −42.1)
Nov	Hispanic	47.8 % (35 to 60.5)	2.1 % (−17.7 to 22)	69.4 % (53 to 85.9)	−27 % (−43.7 to −10.2)	−92.4 % (−116.9 to −67.9)
Feb	Hispanic	10.2 % (4 to 16.5)	−10.5 % (−32.7 to 11.6)	78.8 % (61.5 to 96.2)	−7.1 % (−22.2 to 8.1)	−71.5 % (−86 to −57)
Nov	Asian	75.6 % (63.3 to 88)	−33.7 % (−54 to −13.5)	43.4 % (27.5 to 59.2)	−46.8 % (−74.1 to −19.6)	−38.4 % (−57 to −19.9)
Feb	Asian	79.5 % (67.3 to 91.6)	−42 % (−61.7 to −22.3)	45.2 % (31.2 to 59.2)	−50 % (−68.4 to −31.6)	−32.7 % (−55.2 to −10.3)
Nov	Democrat	43.6 % (40.5 to 46.7)	−15.5 % (−19.3 to −11.6)	44.7 % (41.1 to 48.3)	−24.2 % (−27.6 to −20.8)	−48.7 % (−53 to −44.3)
Nov	Republican	33.5 % (30.7 to 36.3)	−4.5 % (−10.7 to 1.6)	39.7 % (35.9 to 43.5)	−27.1 % (−30.6 to −23.6)	−41.6 % (−46 to −37.2)

Source					
Survey	Group	Government Program or Agency	Economic	Health	Education
Nov	All	−7.8 % (−9.7 to −6)	2.5 % (0.4 to 4.6)	−3.4 % (−5.9 to −0.9)	8.8 % (7.1 to 10.5)
Feb	All	−7.2 % (−9.8 to −4.5)	−9.8 % (−12.7 to −6.9)	6.2 % (3.4 to 8.9)	10.8 % (7.8 to 13.8)
Nov	White	−9.7 % (−12 to −7.3)	1.1 % (−1.8 to 4)	−1 % (−4.4 to 2.3)	9.6 % (7.2 to 12)
Feb	White	4.2 % (−0.2 to 8.6)	−29 % (−33.4 to −24.7)	5.5 % (1.3 to 9.7)	19.3 % (14.6 to 24.1)
Nov	African American	1.3 % (−3.7 to 6.4)	18.8 % (11.6 to 26)	−13.3 % (−21.9 to −4.8)	−6.8 % (−14.1 to 0.5)
Feb	African American	−45 % (−53.4 to −36.6)	24.2 % (11 to 37.4)	7 % (−0.6 to 14.6)	13.8 % (1.1 to 26.5)
Nov	Hispanic	−21.3 % (−28.7 to −13.9)	−30.8 % (−45.8 to −15.9)	−10.5 % (−24.2 to 3.3)	62.6 % (51.4 to 73.8)
Feb	Hispanic	−26.4 % (−35.5 to −17.4)	10.1 % (−4.2 to 24.3)	26.6 % (12.6 to 40.6)	−10.2 % (−27.3 to 6.9)
Nov	Asian	25.1 % (7.8 to 42.3)	−0.3 % (−18.5 to 18)	−15.5 % (−32.1 to 1.1)	−9.3 % (−23.2 to 4.6)
Feb	Asian	−18.6 % (−28.5 to −8.8)	3 % (−22.6 to 28.6)	19.6 % (−3.9 to 43.2)	−4 % (−15.4 to 7.4)
Nov	Democrat	−8.5 % (−11.3 to −5.8)	1.6 % (−1.6 to 4.7)	−4.7 % (−8.5 to −0.8)	11.6 % (8.8 to 14.5)
Nov	Republican	−8 % (−10.7 to −5.3)	2.6 % (−1.1 to 6.2)	−0.4 % (−4.4 to 3.7)	5.9 % (2.9 to 8.8)
